# Feasibility experiment of a novel deformable self-assembled magnetic anastomosis ring (DSAMAR) for gastrointestinal anastomosis through a natural orifice

**DOI:** 10.1038/s41598-024-60887-w

**Published:** 2024-05-08

**Authors:** Miaomiao Zhang, Qiuye Zhong, Jia Ma, Jianqi Mao, Aihua Shi, Yi Lyu, Xiaopeng Yan

**Affiliations:** 1 Department of Hepatobiliary Surgery, The First Affiliated Hospital of Xi’an Jiaotong University, No. 277 West Yanta Road, Xi’an, 710061 Shaanxi China; 2 Shaanxi Provincial Key Laboratory of Magnetic Medicine, The First Affiliated Hospital of Xi’an Jiaotong University, Xi’an, China; 3 National Local Joint Engineering Research Center for Precision Surgery & Regenerative Medicine, The First Affiliated Hospital of Xi’an Jiaotong University, Xi’an, China; 4 Zonglian College, Xi’an Jiaotong University, Xi’an, China; 5 Department of Surgical Oncology, Shaanxi Provincial People’s Hospital, Xi’an, China

**Keywords:** Magnetic surgery, Magnetic compression anastomosis, Magnamosis, Endoscopy, Natural orifice, Gastrointestinal system, Gastrointestinal diseases, Gastrointestinal diseases

## Abstract

Although the application of magnetic compression anastomosis is becoming increasingly widespread, the magnets used in earlier studies were mostly in the shape of a whole ring. Hence, a deformable self-assembled magnetic anastomosis ring (DSAMAR) was designed in this study for gastrointestinal anastomosis. Furthermore, its feasibility was studied using a beagle model. The designed DSAMAR comprised 10 trapezoidal magnetic units. Twelve beagles were used as animal models, and DSAMARs were inserted into the stomach and colon through the mouth and anus, respectively, via endoscopy to achieve gastrocolic magnamosis. Surgical time, number of failed deformations, survival rate of the animals, and the time of magnet discharge were documented. A month later, specimens of the anastomosis were obtained and observed with the naked eye as well as microscopically. In the gastrocolic anastomosis of the 12 beagles, the procedure took 65–120 min. Although a deformation failure occurred during the operation in one of the beagles, it was successful after repositioning. The anastomosis was formed after the magnet fell off 12–18 days after the operation. Naked eye and microscopic observations revealed that the anastomotic specimens obtained 1 month later were well-formed, smooth, and flat. DSAMAR is thus feasible for gastrointestinal anastomosis under full endoscopy via the natural orifice.

## Introduction

Malignant gastric outlet obstruction (MGOO) is a common complication in various malignant tumors, including pancreatic, gastric, ampullary, and biliary tract cancers^1,2^. Symptoms such as nausea, vomiting, abdominal pain, progressive malnutrition, cachexia, and dehydration, which affect patients’ quality of life, are often signs of advanced or metastatic disease. In such cases, only palliative care is indicated^1,3^, with the aim of relieving the symptoms, improving the quality of life, and initiating or resuming systemic chemotherapy^4^. Currently, palliative treatment methods for MGOO include open or laparoscopic gastrojejunostomy (GJ), endoscopic stent (ES) placement, and endoscopic ultrasonography-guided gastroenterostomy (EUS-GE)^5^. However, several investigations have demonstrated that these three treatments share their advantages and disadvantages^4,5^.

In 1978, Obora first proposed the use of magnetic rings for microvascular anastomosis^6^. Owing to advancements in the field, the concepts of magnetic compression anastomosis (MCA)^7^ and magnamosis^8^ were proposed successively. Furthermore, the application scenario of magnetic rings has expanded from vascular anastomosis to gastrointestinal anastomosis. MCA relies on magnetic force to form an anastomosis without the use of sutures or staples^9^. The underlying principle of MCA is that the compressed tissue between two magnets undergoes ischemia, necrosis, and shedding, whereas the adjacent tissue undergoes adhesion, repair, and healing^10^. Presently, the application of MCA in the digestive tract includes esophageal anastomosis^11^, gastrointestinal anastomosis^12^, cholangiojejunostomy^13^, intestinal anastomosis^14^, and colonic anastomosis^15^. These findings from these investigations have established that MCA of the digestive tract is safe and reliable.

To explore a novel palliative treatment method for MGOO, open gastrojejunostomy has previously been performed in rats based on the principle of MCA^16^. For the further investigation of minimally invasive surgical methods, magnets were inserted through the mouth and anus to complete rat gastrocolic anastomosis through the natural lumen^10^. Inspired by previous studies, a deformable self-assembling magnetic anastomosis ring (DSAMAR) suitable for gastrojejunostomy in large animals was designed, and its feasibility was confirmed by performing animal experiments in this study.

## Materials and methods

### The design of DSAMAR

**Figure 1:**
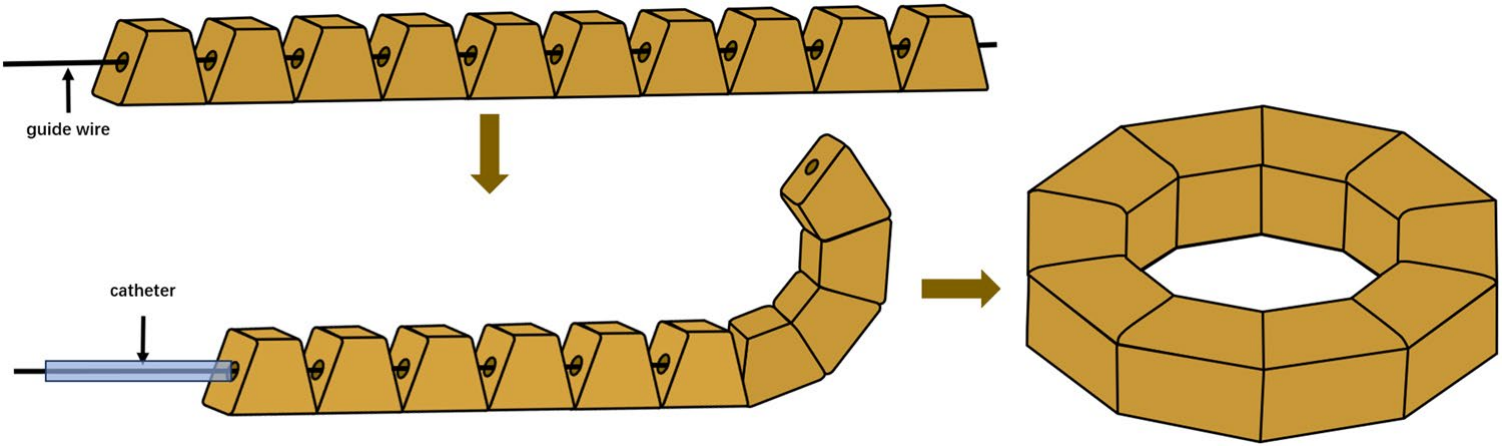
Schematic depiction of DSAMAR.

The DSAMAR comprises 10 magnetic units, and each unit is a trapezoidal magnet with a hexahedral structure. The center of the magnetic unit has a hole along the long axis and is located on the central axis of the left and right sides, which allows the guide wire to pass through it. The 10 magnetic units can be arranged in a straight-chain shape by threading them into the guide wire in the direction opposite to that of the N and S poles of the two adjacent magnetic units. Upon gradually withdrawing the guide wire from the magnetic units by pushing the catheter along the guide wire, the adjacent magnetic units become deformed owing to mutual attraction. When all guide wires are pulled out, all the magnetic units are in turn attracted, which automatically completes the deformation and assembly and results in the formation of a complete magnetic anastomosis ring (Fig. 1).

### Animals

Twelve beagles (male = 6, female = 6, weighting 12–15 kg) were obtained from the Laboratory Animal Center of the Xi’an Jiaotong University. As the aim of the study was to explore the operability of the newly designed magnetic anastomotic rings, all 12 beagles were included in the study group; no control group was maintained. The experimental protocol was approved by the Committee for Ethics of Animal Experiments of Xi’an Jiaotong University (Permit Number: 2022-1451). The research protocol and all experimental procedures complied with the Guidelines for the Care and Use of Experimental Animals, as issued by the Xi’an Jiaotong University Medical Center.

### Surgical procedures for gastrocolostomy

**Figure 2:**
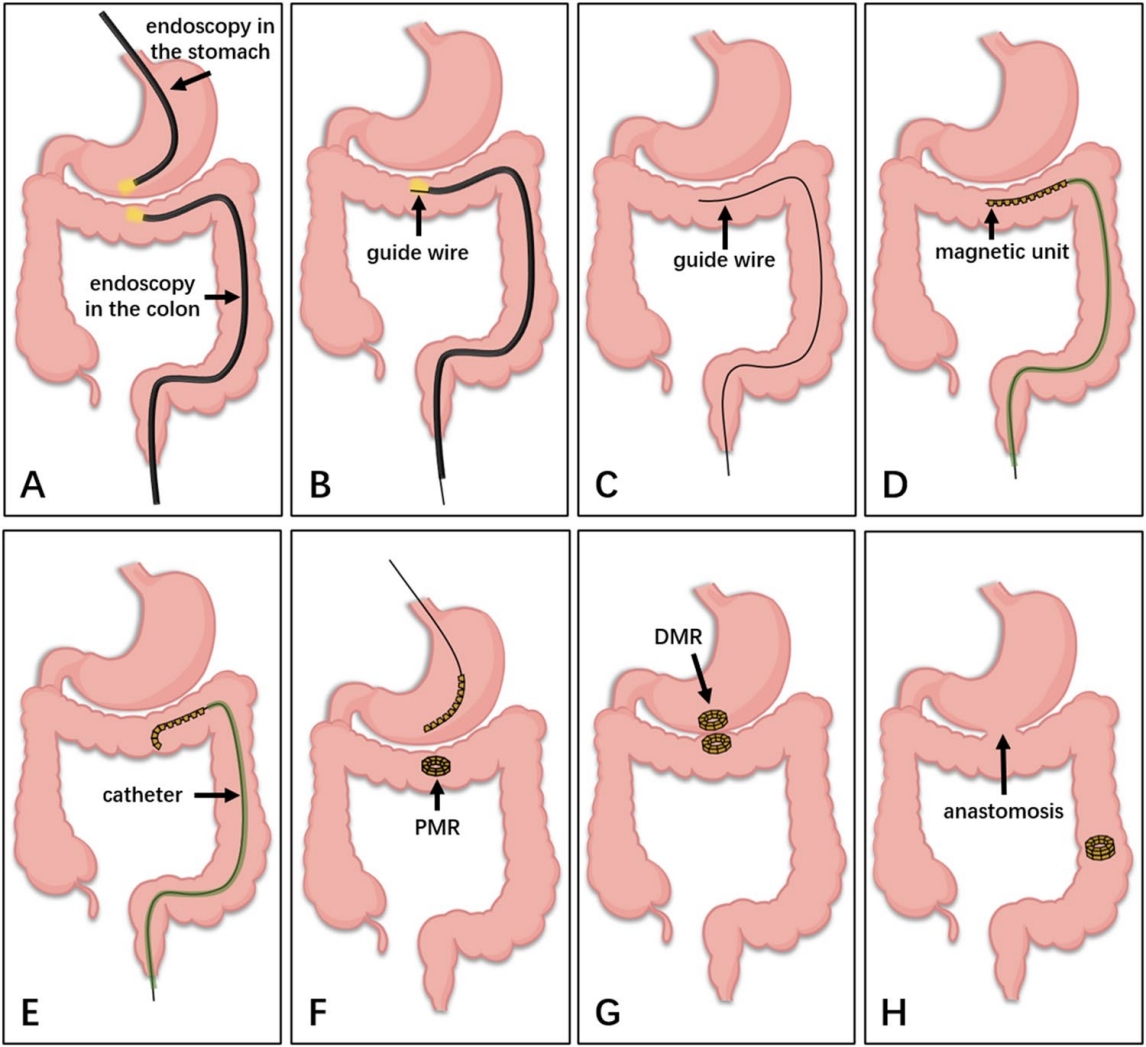
Schematic diagram displaying the gastrocolic anastomosis using DSAMR. (**A**) Two endoscopes were placed through the mouth and anus, respectively, to determine the location for anastomosis. (**B**) The guide wire was inserted through the biopsy hole of the endoscope in the colon. (**C**) The guide wire was fixed and the endoscope was withdrawn. (**D**) The catheter pushed the magnetic units to the guide wire head end. (**E**) As the guide wire exits, the magnetic units gradually deform. (**F**) After the successful deformation of the PMR in the colon, the magnetic units were inserted into the stomach in the same way. (**G**) DMR and PMR attracted together. (**H**) After the magnetic rings fell off, an anastomosis was formed.

All beagles were adaptively fed for 1 week and fasted 12 h before the operation. The animals were anesthetized intravenously with 3% pentobarbital (1 mL/kg) and then fixed on the operating table in the supine position. Two endoscopes were inserted into the stomach through the mouth and into the colon through the anus. Under X-ray monitoring, the head ends of the two endoscopes were placed as close as possible to determine the positions for placing the magnets on both sides (Fig. 2A). The endoscope in the stomach was subsequently removed, following which the guide wire was inserted through the biopsy hole of the endoscope in the colon (Fig. 2B). The position of the head of the guide wire was maintained while withdrawing the endoscope (Fig. 2C). The tail end of the guide wire was inserted successively into the 10 magnetic units, which were pushed to the head end of the guide wire by pushing the catheter (Fig. 2D). The guide wire was gradually extracted while the catheter was being pushed (Fig. 2E). Without the constraint of the guide wire, the magnetic units self-assembled into a ring (Fig. 2F). The magnetic anastomosis ring was similarly placed in the stomach (Fig. 2F,G). The magnetic rings in the colon and stomach were called parent magnetic rings (PMR) and daughter magnetic rings (DMR), respectively. The operation was completed after the two magnetic rings were attracted together (Fig. 2G), and when they fell off, the anastomosis was formed (Fig. 2H).

### Postoperative care

After the surgery and after recovery from anesthesia, the dogs were raised in a single cage and fed normally. The position and status of the magnets were monitored using X-ray.

### Operation and falling-off time of the magnets

The operating time was recorded for each dog’s gastrocolic magnamosis. The falling-off time of the magnets was defined as the time taken for the magnet to be discharged from the body through the anus after the surgery.

### Gross specimen collection

The dogs were euthanized 1 month after the operation using excessive pentobarbital sodium (60 mg/kg). The gastrocolic anastomosis was then removed, including the stomach and approximately 15 cm of the surrounding colon.

### Histological analyses

Anastomotic sections of adequate length were cut and soaked overnight in 10% buffered formalin. After fixation, the anastomosis-bearing segment was embedded in paraffin, and 4 µm–thick sections were cut at the anastomotic site. The sections were later stained with hematoxylin and eosin (HE) and Masson’s trichrome stain and examined under a bright-field microscope.

### ARRIVE guidelines statement

The authors have read the ARRIVE guidelines, and the manuscript was prepared and revised according to the ARRIVE guidelines.

## Results

### Relevant parameters of the animals in experiments

**Figure 3:**
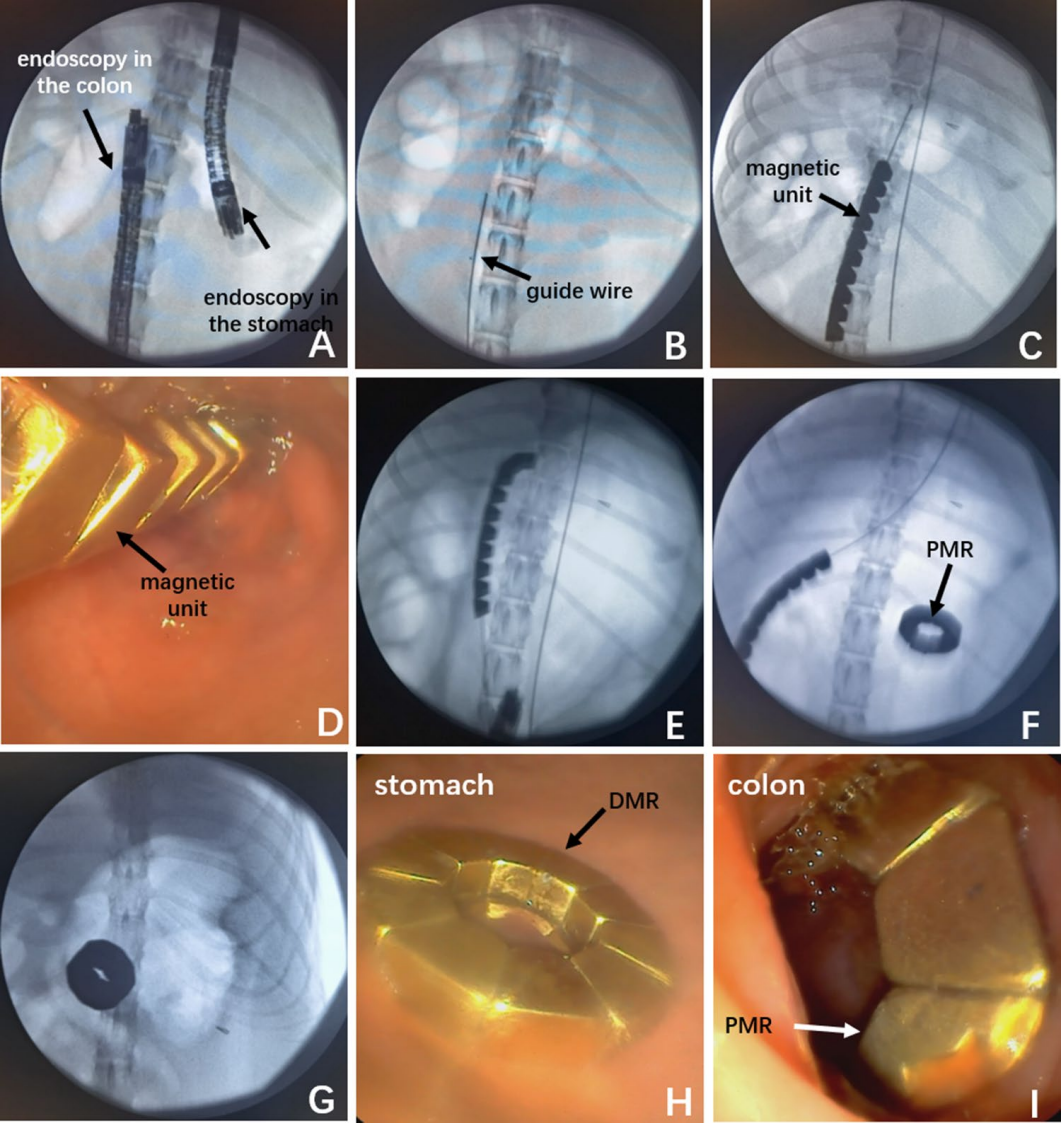
The process of intraoperative operation for a representative dog. (**A**) The specific location to be anastomosed was determined under X-ray monitoring. (**B**) The guide wire in the colon was placed in a suitable position. (**C**) The linear magnetic units in the colon were pushed to the head of the guide wire. (**D**) Magnetic units in the colon were observed through endoscopy. (**E**) The magnetic units in the colon were deforming. (**F**, **G**) After the DSAMAR was placed in the stomach in the same way, the two magnetic rings attracted each other. (**H**) DMR in the stomach was observed by endoscopy. (**I**) PMR in the colon was shown by endoscopy.

During gastrocolic anastomosis in one of the beagles, the magnetic units in the colon failed to deform. Such units were removed and repositioned successfully. Other DSAMARs in the stomach and colon were successfully placed and attracted each other. Thus, gastrocolic magnamosis was successfully established in the 12 beagles (Fig. 3). The surgical duration was 79.92 ± 14.87 min (65–120 min).

### Survival rate and postoperative complications of gastrocolostomy in dogs

All 12 dogs survived after endoscopic gastrocolic magnamosis, and no complications, such as anastomotic leakage or gastrointestinal obstruction, occurred in any of the animals. The magnets fell from the anus 14.83 ± 1.70 days (12–18 days) after the operation.

### Gross and histological appearance of the anastomosis

**Figure 4:**
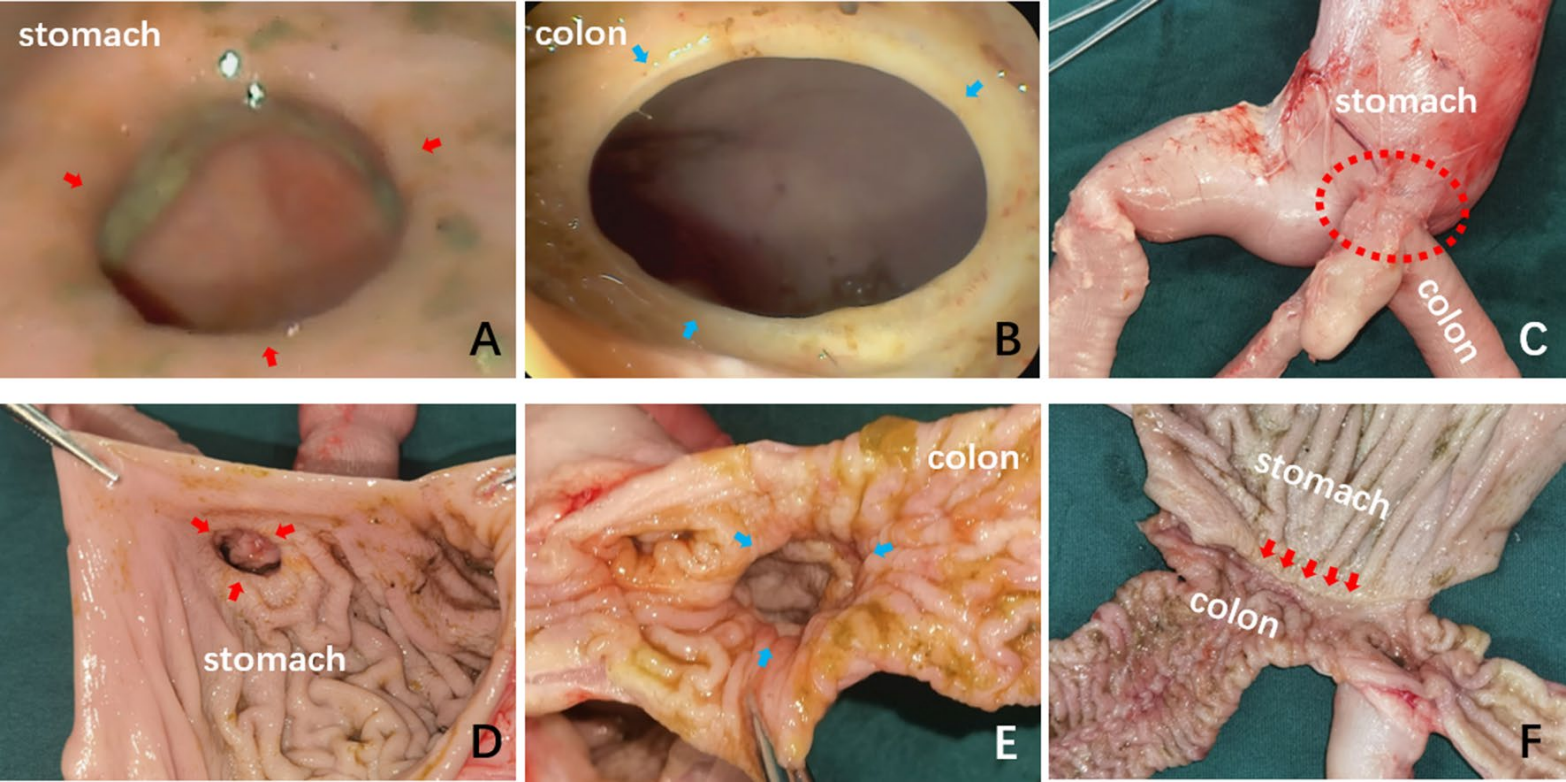
A gross specimen of the anastomotic of a representative dog. (**A**) The anastomosis on the gastric side displayed by an endoscope. (**B**) The anastomosis on the colon was observed by the endoscope. (**C**) The serosal layer of the anastomosis. (**D**) The mucosal layer of the anastomosis was observed from the gastric side. (**E**) The mucosal layer of the anastomosis was observed from the colonic side. (**F**) The mucosal layer of the anastomosis was observed after a longitudinal dissection.

**Figure 5:**
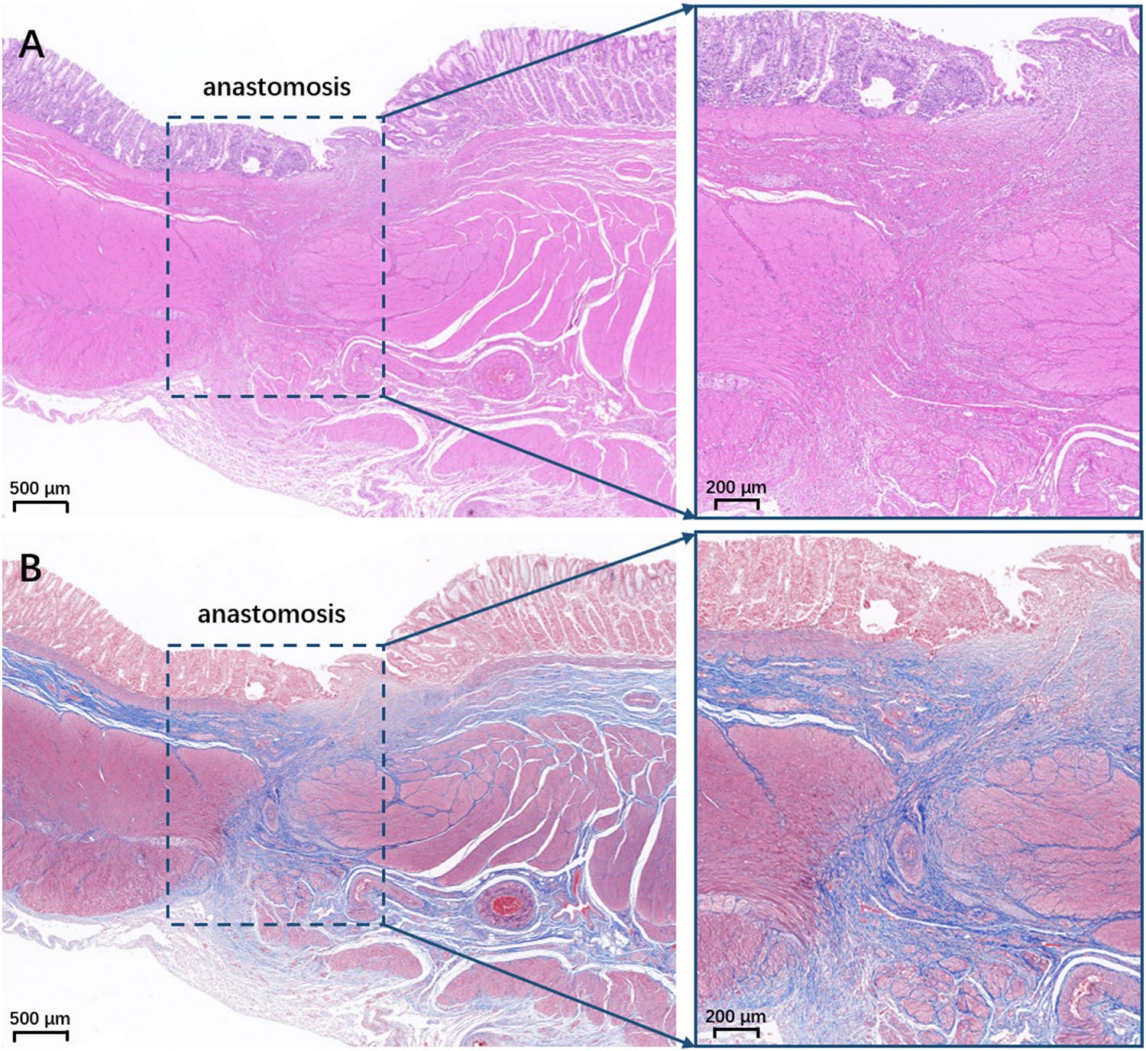
Histological specimen of the anastomosis. (**A**) HE staining (2.0×). (**B**) Masson’s staining (2.0×).

Endoscopy indicated that the anastomosis on the gastric and colonic sides had grown well (Fig. 4A,B). Gross specimens revealed that the serosal and mucosal layers of the anastomosis were smooth and exhibited good continuity (Fig. 4C–F). Furthermore, HE and Masson’s trichrome staining confirmed the continuity of the serosal, submucosal, and mucosal layers (Fig. 5).

## Discussion

The DSAMAR designed in this study assumed a linear state under the constraint of the guide wire, which reached the jejunum through the obstruction of the gastric outlet with endoscopic assistance and then deformed into a ring via self-assembly. The ratio of the cross-sectional area of the linear state to that of the ring formation was approximately 1:15. The design of the DSAMAR was described in detail, and the results showed that the self-assembly and deformation process of the DSAMAR was smooth. In addition, the attraction was smooth, and the anastomosis was formed with good continuity.

In the past, surgical bypass consisting of an open or laparoscopic GJ was the only feasible palliative treatment method available. This technique has been gradually replaced by ES, which had a higher clinical success rate and less trauma burden^17^. Nonetheless, some studies have indicated that stent obstruction caused by tumor ingrowth limits further therapy for MGOO^18^. A recent prospective cohort study reported that EUS-GE demonstrated excellent efficacy in treating MGOO. EUS-GE had an acceptable safety profile and long-term patency and possessed several clinically significant advantages over ES^19^. However, EUS-GE remains a technically difficult and nonstandard therapy and is currently limited to empirical treatment in high-tech centers^5^. DSAMAR is a special magnetic ring designed based on previous studies on MCA and can achieve a larger anastomosis while passing through the obstructed section with a small diameter. In this manner, gastrojejunal anastomosis can be performed entirely via endoscopy. DSAMAR is similar to EUS-GE in that both techniques aim to achieve full endoscopic gastrojejunal anastomosis. Nevertheless, the key difference is that the operating principle of DSAMAR is simple and easy to use. When compared with the surgical procedures and ES placement, DSAMAR can achieve the same effect as the former using a minimally invasive procedure.

Unlike suture and staple anastomosis, magnetic anastomosis is a new “non-penetrating” anastomosis mode. During the former procedure, the tissues at both ends of the anastomosis are subjected to limited “point” forces, whereas, during magnamosis, they are subjected to continuous “surface” forces. Based on these characteristics of magnamosis, it is also referred to as “smart anastomosis”^20^. Some studies have established that magnamosis is associated with less inflammation, scar, distortion, and mural thickening than suture and staple anastomosis owing to the lack of foreign bodies at the anastomotic site^21^. A study has even reported that magnamosis can achieve enteroenterostomy in case of severe peritonitis^15^.

In 2001, Cope et al.^22^ was the first to combine magnetic anastomosis with endoscopic technology to conduct animal experiments on the long-term patency effect of gastrointestinal magnetic anastomosis. The authors applied MCA into clinical practice in 2004^23^. This study employed endoscopic magnetic anastomosis to perform gastric bypass anastomosis in patients with malignant duodenal obstruction. Specifically, the intestinal magnet is placed by delivering the magnet to the intestine using the endoscope after the balloon dilation of the stenosis. A similar clinical study was conducted by an Italian team in 2010, but the study was terminated prematurely after one patient died due to a serious adverse event (i.e., stent perforation)^24^. In 2009, the American scholar Professor Harrison designed a special gradient magnet device and proposed the concept of “Magnamosis” for the first time^8^. Later, Professor Harrison’s team conducted a series of animal experimental studies combining endoscopy techniques with magnamosis^25–27^ so as to achieve the purpose of digestive tract magnamosis in a relatively minimally invasive way. The magnets used in these research were all cylindrical or circular whole magnets, and the whole magnet was easier to insert into the digestive tract under laparotomy or laparoscopy. However, when placing magnets in the digestive tract entirely through endoscopy, if there is a narrowing of the digestive tract, it becomes difficult to place the whole magnets.

The earliest concept of “Self-Assembling” was proposed in 2011. The team of Ryou, an American scholar, designed a self-assembling magnet with a hinged square frame to achieve gastrojejunostomy. The deformation of the magnet was achieved by knotting the suture lines^28^. Next, the team designed a more sophisticated octagonal self-assembling magnet (Incisionless Anastomosis System, IAS) and validated its feasibility in establishing intestinal bypass creation through animal experiments^14,29^. The deformation of IAS needs to be achieved through the biopsy hole of the endoscope; therefore, during the deformation, the endoscope needs to reach the intended site of anastomosis. In cases of gastrointestinal stenosis, if the endoscope cannot pass through the stenosis, it becomes difficult to achieve a full endoscopic magnamosis with IAS. The DSAMAR designed in this study offers unique advantages and is different from the magnets used previously. During the insertion process, DSAMAR decomposes the entire magnamosis ring into 10 magnetic units, which permits them to appear in a linear state under the constraint of the guide wire. Upon reaching the designated anastomotic site, the guide wire is drawn out to make the magnets lose their constraint and gradually absorb and deform into a circular shape. This feature allows DSAMAR to pass through stenosis in a linear state, reach the intended site of anastomosis, and then manipulate it to deform into a circular shape. This feature makes DSAMAR especially conducive for the endoscopic palliative treatment of gastrointestinal obstruction diseases, such as MGOO.

Nonetheless, there are certain limitations in this study. The animal experiments were validated by placing the DSAMARs in the stomach and colon through the oral and anal channels, respectively, to establish gastrocolic anastomosis. This approach preliminarily verified the feasibility of achieving DSAMAR magnamosis completely through the natural lumen. In the next step, an actual gastrojejunostomy should be performed using DSAMAR. In addition, unlike EUS-GE, magnamosis cannot immediately create a gastrojejunal anastomosis. After the magnets on both sides are attracted to each other, the tissue between the two magnets gradually undergoes necrosis. Anastomosis can only be formed after the magnets fall off, which usually takes some time. However, this drawback can be overcome. For scenarios in which instant patency is required, after waiting for the two magnets to attract each other, endoscopic electrocautery can be used to establish an immediate channel in the center of the magnetic rings. Finally, the current version of the DSAMAR is a relatively rough first-generation design; hence, one case of deformation failure was encountered during the actual operation. This failure could be attributed to the fact that the colon was not filled with air and hence did not have enough deformation space, which led to the DSAMAR being subjected to intestinal wall interference during deformation. In the future, the design of the magnetic ring should be refined and more verification studies must be conducted.

## Conclusion

DSAMAR is an ingeniously designed and easy-to-operate magnamosis device, and this study demonstrated the feasibility of performing gastrocolic magnamosis completely via endoscopy. As the next step, the design of the DSAMAR will be improved. More animal experiments can aid in verifying the method’s feasibility and safety, and DSAMAR is expected to achieve clinical application and provide a new minimally invasive therapeutic method for patients with MGOO or other types of gastrointestinal obstruction.

## Data Availability

The datasets used and analyzed during the current study are available from the corresponding author upon reasonable request.
